# Genome-wide association study of copy number variation and early growth traits in inner Mongolian cashmere goats

**DOI:** 10.3389/fvets.2025.1651622

**Published:** 2025-10-17

**Authors:** Yifan Liu, Haijiao Xi, Qi Xu, Bohan Zhou, Jinquan Li, Rui Su, Qi Lv, Yanjun Zhang, Ruijun Wang, Zhiying Wang

**Affiliations:** ^1^College of Animal Science, Inner Mongolia Agricultural University, Hohhot, Inner Mongolia Autonomous Region, China; ^2^Inner Mongolia Key Laboratory of Sheep & Goat Genetics Breeding and Reproduction, Hohhot, Inner Mongolia Autonomous Region, China; ^3^Key Laboratory of Mutton Sheep & Goat Genetics and Breeding, Ministry of Agriculture And Rural Affairs, Hohhot, Inner Mongolia Autonomous Region, China

**Keywords:** copy number variation, CNV-based GWAS, early growth traits, Inner Mongolia cashmere goat, functional genomics

## Abstract

**Introduction:**

The early growth traits including birth weight (BW), weaning weight (WW), pre-weaning average daily gain (ADG) and yearling weight (YW) are crucial productivity indicators that directly influence growth rates of cashmere goats and economic income of herdsmen in the cashmere goat breeding programs. However, the genetic mechanism of these traits in Inner Mongolia Cashmere Goats (IMCGs) has not been elucidated.Copy number variation (CNV), as a prevalent form of genomic structural variation and a significant contributor to the genetic diversity, has emerged as a valuable molecular marker for analysis of complex traits.

**Methods:**

In this study, Whole Genome Sequencing (WGS) data of 461 IMCGs were used to detect CNVs on autosomes and the Genome-Wide Association Study (GWAS) analysis based on CNVs was performed for early growth traits (BW, WW, ADG and YW) of IMCGs.The identified CNVs were further validated through PCR verification. In addition, t-test was performed on the phenotypes of individuals of IMCGs with significant CNVs.

**Results:**

The 26,003 non-redundant CNVs and 5,014 non-redundant CNVRs were detected, covering a total of 1,015.4 Mb (38.97 %) of the autosomal goat genome. The 11 CNVs were significantly associated with early growth traits through GWAS analysis, including two pleiotropic CNVs simultaneously influencing ADG and WW. Through integrated bioinformatics analysis, seven key candidate genes (*ZN845*, *SOX15*, *FGF11*, *GPS2*, *DVL2*, *SPRY4* and *STAT2*) were identified as being associated with early growth traits.Gene ontology (GO) and Kyoto Encyclopedia of Genes and Genomes (KEGG) enrichment analyses demonstrated that these genes were primarily involved in biological pathways related to cell proliferation, differentiation and protein phosphorylation.Among the 11 significant CNVs, 9 CNVs were demonstrated to show significant associations with individual phenotypes.

**Discussion:**

This study significantly expands the genomic CNV map of IMCGs through large-scale genotyping.The findings demonstrate the utility of CNV-based GWAS analysis in elucidating the genetic mechanisms underlying complex traits, providing valuable insights for molecular marker-assisted breeding and molecular genetic research of economically important traits in cashmere goats.

## 1 Introduction

Body weight, as a fundamental growth trait, represents one of the most crucial economic indicators in animal husbandry, with its measurement spanning the entire lifecycle of livestock rearing (1). In goat breeding practices, early growth traits, including birth weight, weaning weight, average daily weight and yearling weight, serve as essential metrics for evaluating caprine growth and development. All of these may directly or indirectly affect other production of goats (2, 3). With the continuous advancement of bioinformatics, numerous studies have firmly established that diverse functional genes play a crucial role in influencing economic traits. However, traditional breeding methods are beset with certain limitations when it comes to delving into the influencing factors at the molecular level. They are unable to swiftly and precisely screen the functional genes closely associated with specific traits.

Genome-wide association Study (GWAS) analysis was first put forward by Risch and Merikangas in 1996 during their research on the genetics of complex human diseases. This innovative approach allows for a more accurate genome-wide exploration and discovery of key genes linked to diseases or traits (4). The superiority of GWAS is manifested in its capability to analyze the associations between genotypes and phenotypes. By conducting association analyses between a large number of genetic markers, such as single nucleotide polymorphisms (SNPs), copy number variations (CNVs), etc., and the phenotypic records of animals, relevant genes that affect some important quantitative economic traits can be identified across the entire animal genome (5–8). Previously, the exploration of genes significantly associated with important economic traits in cashmere goats was mainly conducted through GWAS based on SNPs. This is the first time that GWAS analysis of the early growth traits of Inner Mongolia cashmere goats has been carried out based on CNVs.

Copy number variation generally refers to the gain or loss of genomic segments ranging from 50 base pairs to 5 megabases in length (9). Multiple adjacent and overlapping CNVs can be combined into a copy number variation region (CNVR). Although both CNVs and SNPs can serve as genetic markers in GWAS analysis, compared with SNPs, CNVs can regulate more than five times the number of base pairs, and cover more functional genes (10).

Some studies have demonstrated that CNVs can exert significant impacts on the complex traitss and disease resistance of individuals by regulating the expression levels of functional genes related to relevant traits, and play a crucial role in the evolutionary process (11). Xu et al. detected CNVs using the aCGH method and subsequently conducted GWAS. It was discovered that the CNV of the *MYH3* gene significantly impacts skeletal muscle development in both Nanyang and Qinchuan cattle (12). Liang et al. found that the CNV of *SERPINA3-1* gene and *GAL3ST1* gene in Qinchuan cattle also affected bovine muscle development (13). Ke et al. observed that when the CNV type of the *PLEC* gene on chromosome 14 of Leizhou black goats is in the amplified state, the chest circumference, body weight, carcass weight, cross - sectional area of the longissimus dorsi muscle, and shear force of individuals with the amplified CNV are superior to those of individuals with other CNV types (14). Wang et al. identified a 2,800 - bp CNV in the *ORMDL1* gene of sheep, which has a significant effect on the body height, chest circumference, body weight, withers height, and cannon circumference of sheep (*p < 0.05*) (15). Feng et al. found that the CNV of *PIGY* gene on chromosome 6 of sheep had a significant effect on the body weight, chest circumference and cannon circumference of sheep (*p < 0.05*), and the amplified type of CNV had a positive impact on the body weight of sheep (16). Qiu et al. discovered that the CNV of *PDGFA*, *GPER1* and other genes in American Duroc pigs could affect growth traits, such as daily weight gain and slaughter weight (17).

Inner Mongolia cashmere goat is a characteristic breed of Inner Mongolia Autonomous Region in China, enjoying a high reputation worldwide. The cashmere is known as “soft gold,” and the mutton is praised as “ginseng in meat,” serving as an important source of economic income for local herders. Currently, there are numerous relevant studies on GWAS using CNV as a genetic marker. However, there has been no related research on conducting GWAS analysis for the early growth traits of Inner Mongolia cashmere goats based on CNV. In this study, the 10X resequencing data of 461 Inner Mongolia cashmere goats, the phenotypic data of early growth traits, and the systematic environmental effect data of IMCGs were used to carry out GWAS analysis based on CNV with mixed linear model. The aim of this study is to detect significant CNVs and related functional genes that affect the early growth traits, providing a theoretical basis for the genetic improvement of the production performance of Inner Mongolia cashmere goats.

## 2 Materials and methods

### 2.1 Ethical approval

In the experiment, the breeding environment complied with the relevant standards for general animal experimental facilities in the Chinese national standard “Laboratory Animal Environment and Facilities” (GB 14925–2023). The feeding and experimental operations of the animals met the requirements of animal welfare. In this study, no anesthesia or euthanasia was performed on experimental animals.

### 2.2 Phenotypic measurement and sample preparation

The Inner Mongolia cashmere goats (*n* = 461) used in this study were all obtained from Inner Mongolia Yiwei White Cashmere Goat Co., Ltd. All the goats were reared under consistent feeding environments and nutritional conditions, being provided with the same commercial diet and having unrestricted access to water. All Inner Mongolia cashmere goats used in this study were born from 2010 to 2018, and all of the data were recorded in detail. Before weaning, some nutritional supplements were provided in addition to the ewe’s milk. The lambs were weaned when they were 4 months old. Birth weight (BW), weaning weight (WW) and weight at 12 months (YW) were measured using electronic scales in the same environment. Additionally, the weaning date was recorded. Average daily gain before weaning (ADG) was calculated as (weaning weight - birth weight) /days to weaning. BW was measured 0.5 h after birth, and WW and YW were measured 12 h after fasting (2). Ear tissue samples were carefully collected using ear-notch forceps. Immediately after collection, they were swiftly transferred into pre-prepared cryotubes filled with 75% alcohol. Subsequently, these cryotubes were stored at −80°C until DNA extraction was carried out.

### 2.3 Whole genome resequencing

Total Genomic DNA was extracted from ear tissue using the traditional phenol-chloroform method, yielding a concentration of 50 ng/μL. The quality of DNA in all samples (461 DNA samples) was evaluated based on light absorption ratios (A260 / 280 and A260/230) and gel electrophoresis (18). Following DNA extraction, qualified samples were fragmented using a Covaris crusher. The DNA fragments were then end-repaired, polyA-tailed, ligated with sequencing adapters, purified, and PCR-amplified to construct the sequencing library. The library was preliminarily quantified using Qubit 3.0, with insert size distribution verified by the Agilent 5,300 system. After passing quality control, the library was subjected to paired-end sequencing (PE150) on the DNBSEQ-T7 platform.

### 2.4 Copy number variation segmentation and genotyping

The raw data were disconnected, filtered and subjected to various quality control steps to obtain clean data for subsequent bioinformatics analysis. In order to ensure the accuracy of the analysis, FastQC v0.11.5 was used to perform strict quality inspection on the original reading length (19). The original readings were then compared with the *Capra hircus* reference genome (Genome assembly ASM4082201v1)^1^ using the Burrows-Wheeler Aligner (BWA aln) v0.7.8 with default parameters (20). On average, 99% of the reads were successfully mapped to the reference genome, achieving a final average sequencing coverage of 9.46 × (ranging from 7 × to 12×) per individual. The initial BAM files, containing sequence alignment data for each individual, were generated using BWA and subsequently sorted and indexed using SAMtools v1.0 (21). Then the genome features such as GC content, repeat and gap content, read count and absolute copy number were calculated using the sliding window method (1 kb window, 800 bp step) (22). For the initial CNVnator output, quality control was performed by filtering copy number variations (CNVs) based on length (50 bp to 5 Mb). Further CNVs filtering was performed using Plink v1.90 beta to remove CNVs with minor allele frequency (MAF < 0.01). CNV regions (CNVRs) were defined by merging quality-controlled CNVs with ≥1 bp overlap at identical genomic positions across all samples using HandyCNV (R v4.4.1). HandyCNV defines CNVR as three types: amplification, deletion and mixing (when deletion and amplification occur in the same region) (23).

### 2.5 Genome-wide association study

The general linear model was used to perform the GWAS analysis based on CNV for early growth traits in IMCGs with Plink v1.90 software. The formula for this model is as follows:


y=Xα+Zβ+e.

Where y is the phenotype vector, α is the fixed effect vector, the fixed effects included herds, sex, maternal age, and year of measurement. X is the structure matrix of fixed effect. β is CNV effect, Z is the structure matrix of CNV effect; e is the residual effect, and the distribution is $e\sim N(0,\sigma^{2})$ .

In the CNV-based GWAS, the Bonferroni method was used to determine the genome-wide significant (0.05/N) threshold, where N represents the number of CNVs. Given that is a stringent criterion, a more lenient threshold was also used for detecting the suggestive (1/N) CNVs (24, 25). The qqman package in R software was used to plot the Manhattan and Q-Q plots (26).

### 2.6 Validation of CNV by qPCR

Eight candidate CNVs associated with early growth traits in IMCGs were selected for validation, including two for BW traits (CNV_DEL_17406 and CNV_DEL_18821), three for ADG and WW traits (CNV_DEL_11189, CNV_DEL_17895, and CNV_DUP_18956), and three for YW traits (CNV_DEL_4359, CNV_DEL_4552, and CNV_DUP_20170). These CNVs were subsequently verified using real-time quantitative polymerase chain reaction (qPCR). The primers for these eight CNVs were designed using Primer Premier 5 software (Supplementary Table S1). Following the methodology established by Sonika et al. (27), the *ACTB* were selected as the reference gene, because the gene is highly conserved in goats and exists in the reference genome in the form of a single copy. A total of 64 DNA samples were selected for verification, including 32 samples containing the target CNV, and 32 normal samples without copy number variation in the test area were used as controls. The qPCR experiment was conducted using the 2 × SG Green qPCR Mix (with ROX; SINOGENE, Beijing, China). The PCR reaction was performed in a total volume of 15 μL, containing 1 μL DNA template (50 ng/μL), 0.25 μL each of forward and reverse primers (10 pM/μL), 5 μL of 2 × Blue-SYBR Green mixture, and 6 μL of nuclease-free water. The thermal cycling protocol consisted of an initial denaturation at 95°C for 10 min, followed by 40 cycles of amplification (95°C for 20 s, 60°C for 30 s), and a final dissociation curve analysis (95°C for 15 s, 60°C for 30 s, and 95°C for 15 s). All reactions were carried out in triplicate on a 96-well transparent reaction plate, and the average cycle threshold (Ct) values were calculated for subsequent copy number analysis. The relative copy number variation in the target region was determined using the 2(^-ΔΔCt^) method, where ΔΔCt was calculated as follows: [(mean Ct of target gene in test sample)–(mean Ct of reference gene (GCG) in test sample)]–[(mean Ct of target gene in reference sample)–(mean Ct of GCG in reference sample)]. Based on this calculation, a value approximating 2 was considered normal, while values ≥ 3 and ≤ 1 indicated copy number gain and loss, respectively.

After using qPCR to verify CNVs, the phenotypic data of individuals with significant CNVs in GWAS analysis were tested by t-test to verify the authenticity of GWAS results.

### 2.7 Candidate gene annotation and functional enrichment analysis

The physical position information was retrieved from the *Capra hircus* reference genome (Genome assembly ASM4082201v1, see Footnote 1). Candidate genes located within 300 kb upstream and downstream of the significant CNVs were identified using the bedtools software (28). Subsequently, the overlapping genes were subjected to enrichment analysis using the Database for Annotation, Visualization, and Integrated Discovery (DAVID) tool, which included Gene Ontology (GO) functional annotation and Kyoto Encyclopedia of Genes and Genomes (KEGG) enrichment analyses. Enrichment terms with statistical significance (*p <* 0.05), determined by Fisher’s exact test, were selected to further investigate genes involved in relevant biological pathways and processes (17, 29). Gene functions were queried using Ensembl Biomart.^2^

## 3 Results

### 3.1 Phenotypic statistics of early growth traits in IMCGs

**Figure 1:**
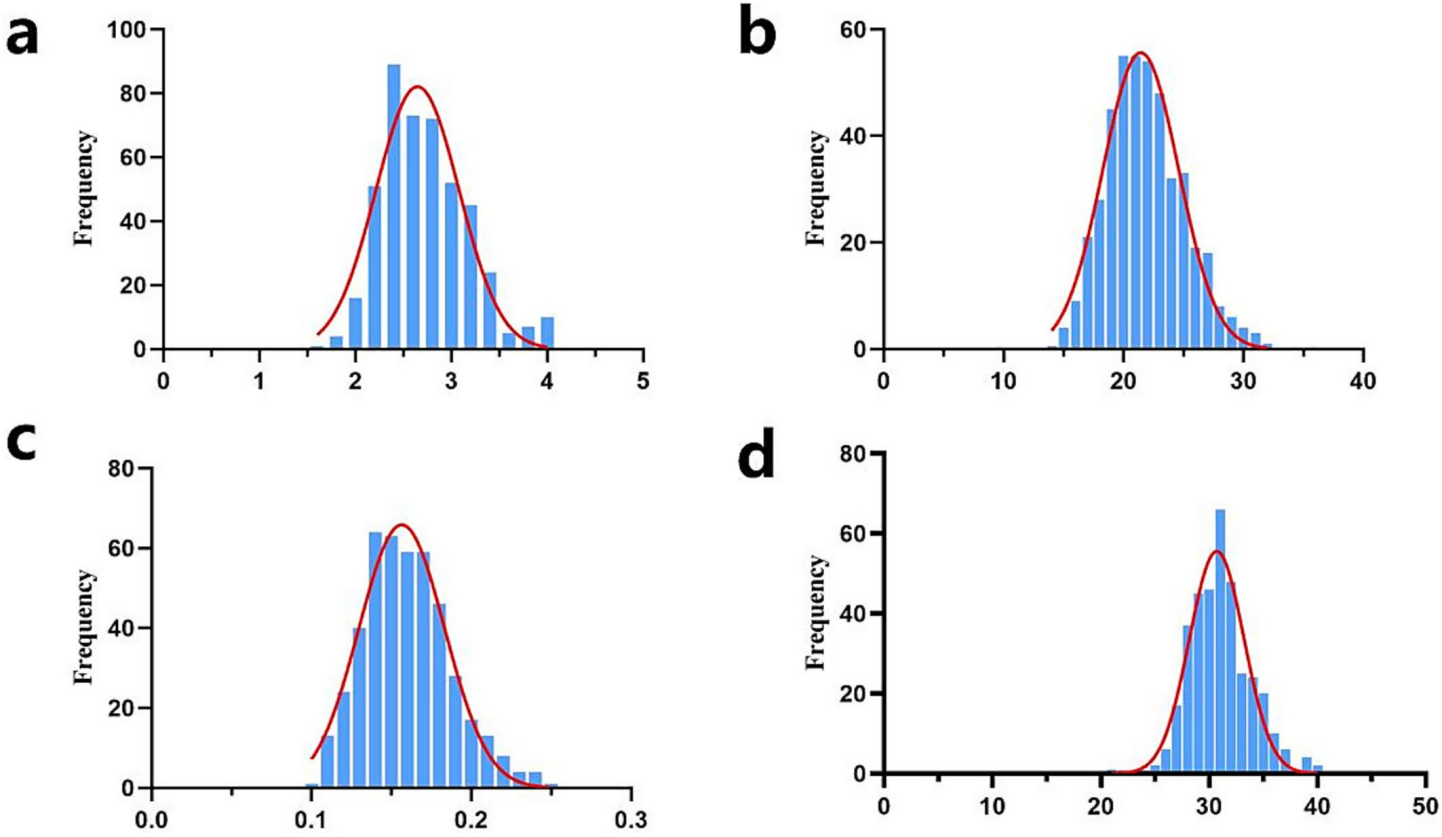
Normal distribution diagram of early growth traits of Inner Mongolia cashmere goats. **(a)** Normal distribution map of birth weight traits (unit: kg); **(b)** Normal distribution map of weaning weight traits (unit kg); **(c)** Normal distribution map of average daily gain (unit: kg); **(d)** Normal distribution of yearling weight traits (unit: kg); frequency, the frequency of different phenotypic values.

In this study, the descriptive statistics of our early growth traits, including birth weight (BW), weaning weight (WW), average daily gain (ADG), and yearling weight (YW), were summarized in Table 1. The mean values (±SD) were determined as 2.66 ± 0.45 kg for BW, 21.90 ± 3.28 kg for WW, 0.16 ± 0.03 kg for ADG, and 31.21 ± 3.60 kg for YW. The coefficients of variation (CV) for the four traits were calculated at 17.10, 14.98, 18.75, and 11.53%, respectively. Prior to statistical analysis, data quality control were implemented, including the exclusion of phenotypic records with missing values and the removal of outliers exceeding the threshold of mean ± 3 standard deviations. Consequently, the sample sizes presented in Table 1 for each trait are slightly reduced from the initial cohort of 461 individuals. Furthermore, normality assessment through statistical tests confirmed that all traits followed a normal distribution pattern (Figure 1), validating the suitability of parametric statistical methods for subsequent analyses.

**Table 1:** Descriptive statistics of early growth traits of IMCGs (unit: kg).

Trait	Number	Mean	SD	Max	Min	CV(%)
BW	448	2.69	0.46	4.00	1.70	17.10
WW	443	21.90	3.28	31.73	14.86	14.98
ADG	443	0.16	0.03	0.246	0.103	18.75
YW	359	31.21	3.60	39.5	21	11.53

BW, birth weight; WW, weaning weight; ADG, average daily gain; YW, yearling weight; SD, standard deviation; Max, maximum; Min, minimum; CV, coefficient of variation.

### 3.2 Detection of genome-wide copy number variation in IMCGs

**Figure 2:**
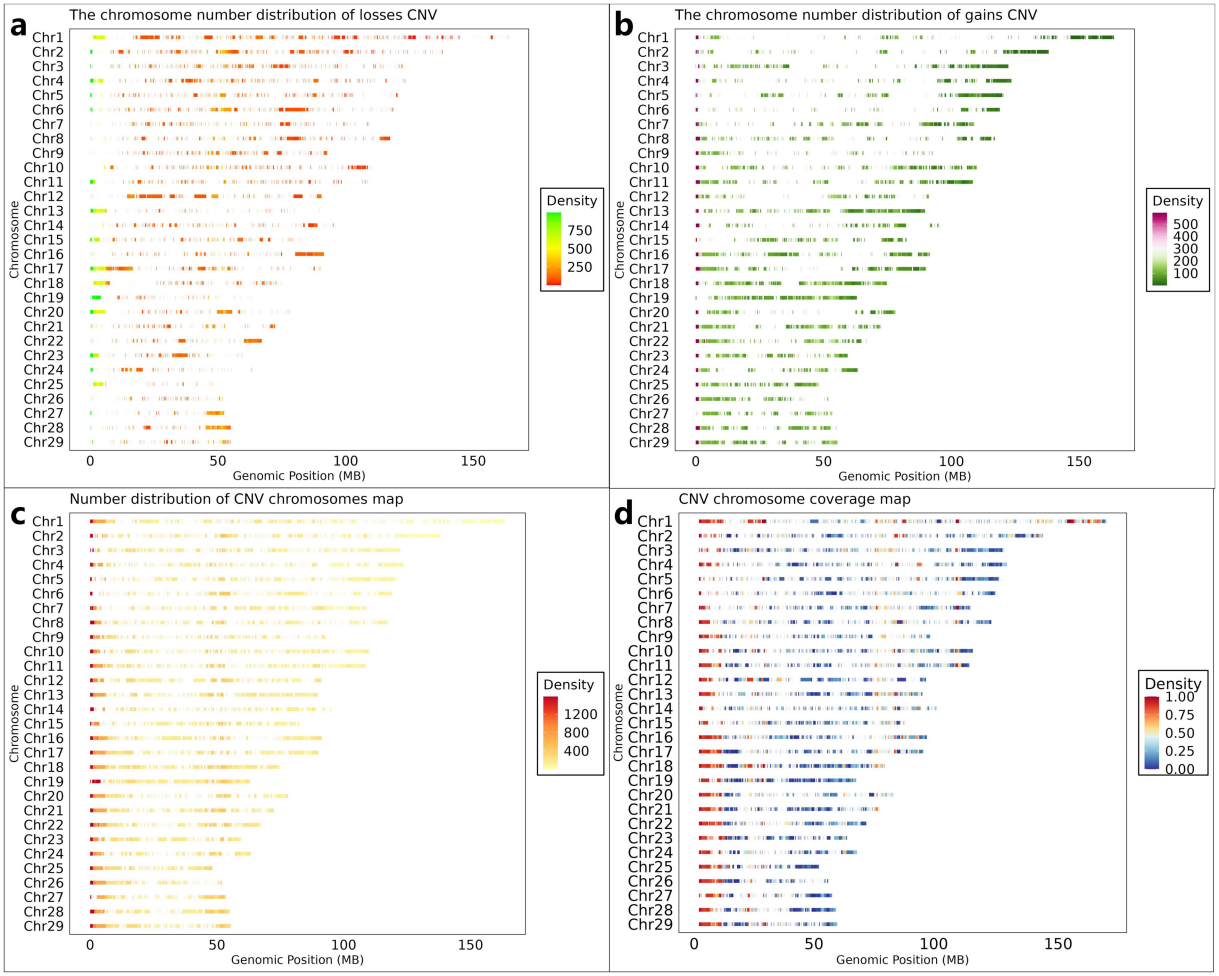
Genome-wide CNV distribution characteristics in IMCGs. **(a)** The chromosome density distribution of the gains CNV; **(b)** The chromosome density distribution of the losses CNV; **(c)** The distribution of the number of CNVs on each chromosome; **(d)** The distribution of the density of CNVs on each chromosome after dividing the area according to the size of 1 Mb. Chr, chromosome; Genomic Position, the position of CNV on the chromosome.

Following quality control procedures, 11.87 TB of high-quality reads were retained from the initial 13.41 TB raw sequencing data obtained from 461 Inner Mongolian Cashmere Goats (IMCGs). Genome-wide CNV detection was performed across all 29 autosomes using CNVnator (v0.4.1). The initial analysis identified 315,891 CNV events, categorized as either losses (copy number = 0 or 1) or gains (copy number ≥ 2). To ensure data reliability, stringent filtering criteria were implemented. CNVs with population frequencies below 0.01 (observed in fewer than 5 individuals) were excluded as potential false positives. Furthermore, overlapping CNVs detected at identical genomic positions across multiple individuals were merged and systematically reannotated. This rigorous filtering process yielded a final set of 26,003 non-redundant CNVs, comprising 10,216 gain-type and 15,787 loss-type events (Figures 2a,b), each present in at least five individuals. The cumulative length of identified CNVs spanned 3,469.5 Mb, exceeding the total length of the goat autosomes (2,605.7 Mb). This apparent discrepancy arises from the overlapping nature of CNVs across individuals during the detection process, preventing direct calculation of chromosomal coverage. To address this, we generated two complementary visual representations: illustrates the density distribution of CNVs across chromosomes was shown in Figure 2c, while Figure 2d presents CNV coverage using a 1 Mb sliding window approach along the chromosomes.

The genomic distribution and characteristics of CNVs across the 29 autosomes are summarized in Table 2. On average, each chromosome contained 897 CNVs, consisting of 352 gain-type and 545 loss-type events. While CNV distribution generally correlated with chromosome length, notable exceptions to this trend were observed. Chromosome 17 exhibited the highest CNV count (1,638). In contrast, chromosome 26 showed the lowest CNV frequency (359).

**Table 2:** The distribution of all CNVs in the 29 autosomes of IMCGs.

Chr	CNVs counts	DUP_CNVs counts	Length of DUP_CNVs (kb)	Max size (kb)	Average size (kb)	Min size (kb)	DEL_CNVs counts	Length of DEL_CNVs (kb)	Max size (kb)	Average size (kb)	Min size (kb)
1	1,503	415	73,980	3,422	178	5.6	1,088	93,020	4,050	85	1.6
2	817	324	72,388	4,587	223	11.2	493	48,960	2,739	99	1.6
3	1,036	502	88,685	3,294	177	7.2	534	33,659	1,426	63	1.6
4	827	328	63,679	2,690	194	9.6	499	40,498	2,582	81	1.6
5	846	338	96,786	4,485	286	4.8	508	26,974	2,286	53	1.6
6	633	174	33,754	3,142	194	12.8	459	40,347	4,634	88	1.6
7	687	330	84,095	3,285	255	11.2	357	18,855	1778	53	1.6
8	880	352	64,792	3,549	184	7.2	528	12,378	726	35	1.6
9	655	214	31,962	2,146	149	8.8	441	34,011	1710	77	1.6
10	1,203	705	97,979	2,422	139	8.0	498	33,174	1,249	67	0.8
11	909	390	117,598	3,729	302	6.4	519	27,412	1,414	53	1.6
12	720	258	46,924	4,130	182	10.4	462	87,199	4,165	189	1.6
13	1,310	378	125,859	4,086	333	9.6	932	58,391	1,208	63	1.6
14	491	209	47,794	4,354	229	8.8	282	24,636	3,558	87	1.6
15	715	362	70,346	1770	194	8.8	353	22,931	2049	65	1.6
16	1,229	559	121,854	4,996	218	5.6	670	2,281	4,857	73	1.6
17	1,638	500	106,091	4,653	212	8.0	1,138	114,083	2,491	100	1.6
18	1,493	629	137,963	4,555	219	7.2	864	55,394	1,189	64	1.6
19	609	374	113,817	4,564	304	11.2	235	10,993	3,342	47	0.8
20	811	157	40,919	2086	261	8.0	654	79,986	2,543	122	1.6
21	924	405	93,685	2,100	231	9.6	519	24,792	1,257	48	0.8
22	905	268	70,214	3,579	262	13.6	637	63,890	2,682	100	1.6
23	880	281	71,557	3,488	255	9.6	599	55,915	2,194	93	1.6
24	391	192	45,366	4,572	236	9.6	199	11,270	2,479	57	1.6
25	755	326	91,760	4,768	281	7.2	429	27,846	1980	65	1.6
26	359	194	56,654	4,493	292	9.6	165	6,951	670	42	1.6
27	948	280	46,586	2,906	166	8.0	668	51,005	1,039	76	1.6
28	1,031	328	74,822	3,056	228	7.2	703	64,270	1950	91	1.6
29	798	444	98,039	4,518	221	8.8	354	12,378	726	35	0.8
total	26,003	10,216	2,285,946	224			15,787	1,183,502	75		

Chr, chromosome; CNVs counts, number of gain-type CNVs + number of loss-type CNVs; DUP_CNVs counts, number of gain-type CNVs; Length of DUP_CNVs, the total length of gain-type CNV; DEL_CNVs counts, number of loss-type CNVs; Length of DEL_CNVs, the total length of loss-type CNVs; Max size, the maximum value of different types of CNV; Average size, the average value of different types of CNV; Min size, the minimum value of different types of CNVs.

The identified CNVs exhibited a size range from 0.8 kb to 4.99 Mb, with a mean length of 133 kb. Specifically, the largest CNV identified was CNV_DUP_13234 located on chromosome 16, spanning 4.99 Mb, while the smallest was CNV_DEL_7892 on chromosome 10, measuring 0.8 kb.

Of the total 26,003 CNVs detected, gain-type CNVs accounted for 39.3% (*n* = 10,216), ranging in size from 4.8 kb to 4.99 Mb. These gain events collectively spanned 2,285.9 Mb, with an average length of 224 kb. In contrast, loss-type CNVs represented 60.7% (*n* = 15,787), ranging from 0.8 kb to 4.85 Mb in length. The cumulative length of deletion CNVs was 1,183.5 Mb, with an average size of 75 kb.

**Figure 3:**
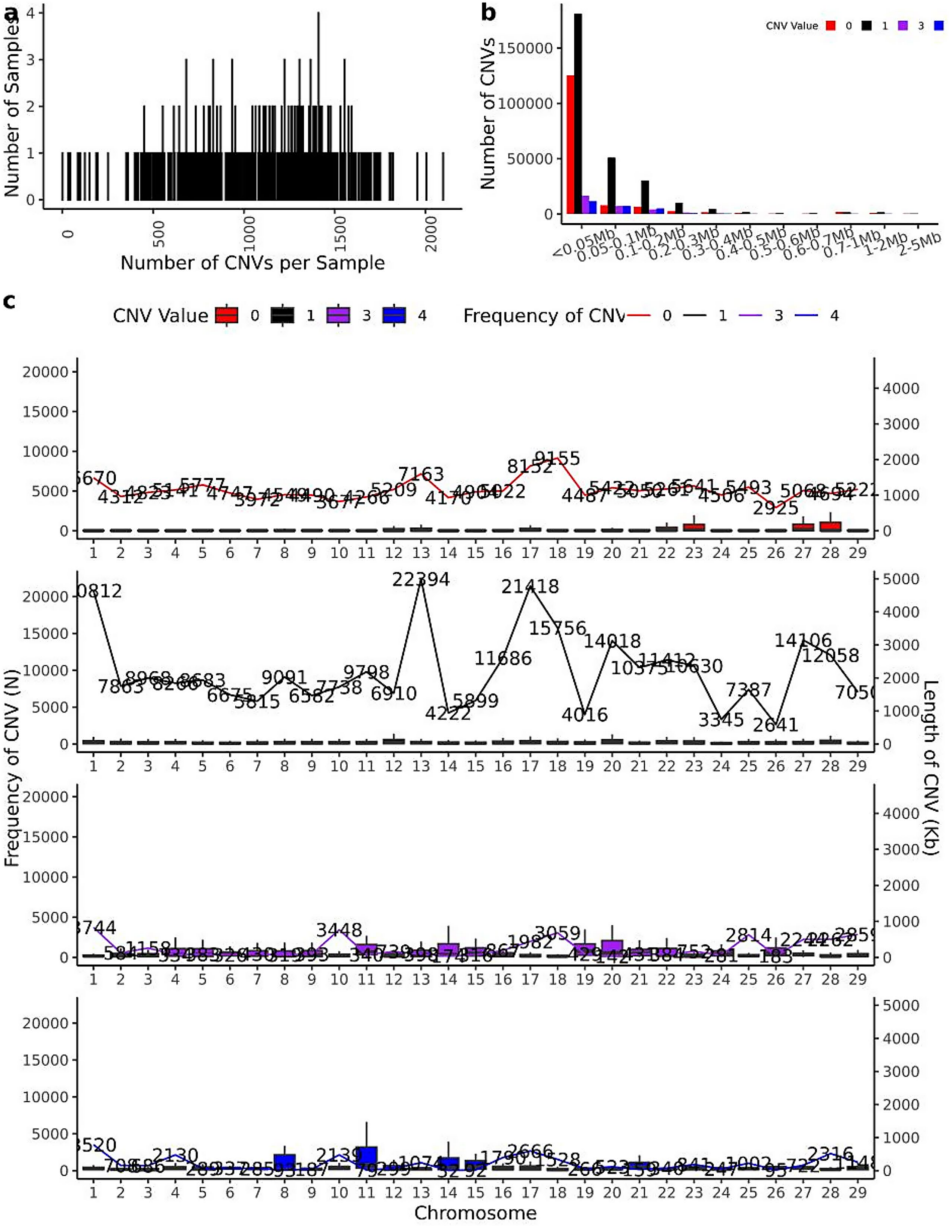
CNV mathematical statistics diagram. **(a)** The statistical map of the number distribution of CNVs in 461 individuals; **(b)** the statistical map of the length of CNVs in 461 individuals (where CNVs with copy number greater than 4 are counted as copy number 4); **(c)** The distribution map of CNVs on chromosomes of 461 individuals (where CNVs with copy number greater than 4 are counted as copy number 4).

The CNV distribution patterns across chromosomes in 461 IMCG individuals are illustrated in Figure 3, where copy numbers of 0 or 1 represent losses and 3 or 4 represent gains. The individual CNV count distribution, revealing that the top three individuals contained 2,097, 2,004, and 1,955 CNVs respectively, while the bottom three individuals exhibited only 43, 34, and 31 CNVs. The average population was 685 CNVs per individual (Figure 3a). The size distribution of CNVs across different copy number categories was shown in Figure 3b. The majority of CNVs (less than 0.05 Mb) were significantly more abundant than larger CNVs (2–5 Mb). Loss-type CNVs (copy number = 0 or 1) substantially outnumbered gain-type CNVs (copy number = 3 or 4), with most CNVs concentrated below 0.3 Mb in length.

Chromosomal distribution patterns are shown in Figure 3c. Loss-type CNVs (copy number = 0) were most prevalent on chromosome 18 (9,155 events) and least frequent on chromosome 26 (2,925 events). Similarly, CNVs with copy number = 1 showed maximal distribution on chromosome 13 (22,394 events) and minimal on chromosome 26 (2,641 events). For gain-type CNVs, those with copy number = 3 were most abundant on chromosome 10 (3,448 events) and least on chromosome 26 (183 events), while copy number = 4 CNVs peaked on chromosome 1 (3,520 events) and were rarest on chromosome 14 (32 events). This chromosomal distribution analysis confirms the relatively low CNV frequency on chromosome 26, consistent with the merged and reannotated CNV data presented in Table 2. The observed patterns suggest potential chromosome-specific mechanisms influencing CNV formation and maintenance in IMCGs.

Through integration of the 26,003 quality-controlled CNVs, we identified 5,014 CNV regions (CNVRs; Supplementary Table 2) by merging adjacent CNVs with overlapping regions exceeding 1 bp. These CNVRs spanned all autosomes, ranging from 75 to 344 regions per chromosome, with a cumulative length of 1,015.43 Mb, representing 38.97% of the goat autosomal genome.

The CNVRs were classified into three categories: 1,085 gain-type CNVRs with a total length of 175 Mb (average length: 161.3 kb), 3,406 loss-type CNVRs spanning 328.76 Mb (average length: 96.5 kb), and 523 mixed-type CNVRs covering 511.67 Mb (average length: 978.3 kb). Chromosome 19 exhibited the highest CNVR coverage (65.58%), while chromosome 9 showed the lowest coverage (27.20%).

Analysis of the 20 longest CNVRs revealed that 50% were mixed-type, with 6 located in chromosomal telomeric regions. This distribution pattern suggests that CNVs frequently occur in highly repetitive telomeric regions, which are known hotspots for large-scale genomic recombination and replication events.

### 3.3 GWAS of copy number variations with phenotype of early growth traits in IMCGs

To investigate the functional significance of CNVs in goat growth development, Genome-wide association studies (GWAS) were conducted for four early growth traits to investigate the functional significance of CNVs in goat growth development. The results are presented in Table 3. Using the Bonferroni correction method, A genome-wide significance threshold of 3.85 × 10^−5^ (corresponding to 1/N, where *N* = 26,003) was established for identifying significant CNV-trait associations. The GWAS analysis revealed 11 CNVs that surpassed the significance threshold, demonstrating associations with early growth traits. Specifically, two genome-wide significant CNVs associated with BW on chromosomes 18 and 20, three CNVs associated with both WW and ADG on chromosomes 13, 19, and 20, and six CNVs associated with YW distributed across chromosomes 3, 5, 21, 22 and 23 were identified. Consistent with the previously reported high genetic correlation between WW and ADG, our analysis identified two overlapping CNVs (CNV_DEL_11189 and CNV_DEL_17895) that showed significant associations with both traits. These CNV regions, along with all other identified variants, were precisely mapped and validated using bedtools software. Functional annotation of the 11 significant CNVs revealed their association with 91 protein-coding genes, as detailed in Table 4.

**Table 3:** Significant CNV descriptive statistics.

Trait	CNV ID	Type	Chr	Start(bp)	End(bp)	*p-*value	Candidate genes
BW	CNV_DEL_17406	DEL	18	67,870,401	67,903,200	3.04E-06	*ENK25, ZN**845**, ZN160, ZN208*
	CNV_DEL_18821	DEL	20	22,544,801	22,552,000	1.18E-05	
WW	CNV_DEL_11189	DEL	13	3,608,001	4,816,000	7.61E-06	*TRA2B*
	CNV_DEL_17895	DEL	19	27,445,601	27,462,400	1.76E-05	*KCD11, SOX**15**, GBRAP, S35G3, TMM95, IPP2, SPEM1, CD68, SPEM2, SAT2, RNK, GPS**2**, CLD7, MPU1, TM102, BAP18, TNF13, TM256, MOT13, ZBTB4, ASGR1, PHF23, SHBG, CNEP1, PLS3, YBOX2, ACADV, IF5A1, IF4A1, ELP5, EFNB3, GLUT4, FGF**11**, BCL6B, DVL**2**, TNF12, TNK1, ACHB, SENP3, NEUL4, ACAP1, TCAB1, ASGR2, P53, FXR2, LOX12, NLGN2, DLG4, RPB1, DYH2*
ADG	CNV_DEL_11189	DEL	13	3,608,001	4,816,000	7.61E-06	*TRA2B*
	CNV_DEL_17895	DEL	19	27,445,601	27,462,400	1.76E-05	*KCD11, SOX**15**, GBRAP, S35G3, TMM95, IPP2, SPEM1, CD68, SPEM2, SAT2, RNK, GPS**2**, CLD7, MPU1, TM102, BAP18, TNF13, TM256, MOT13, ZBTB4, ASGR1, PHF23, SHBG, CNEP1, PLS3, YBOX2, ACADV, IF5A1, IF4A1, ELP5, EFNB3, GLUT4, FGF**11**, BCL6B, DVL**2**, TNF12, TNK1, ACHB, SENP3, NEUL4, ACAP1, TCAB1, ASGR2, P53, FXR2, LOX12, NLGN2, DLG4, RPB1, DYH2*
	CNV_DUP_18956	DUP	20	69,328,801	69,360,800	2.55E-05	*TCTP, TCPE, ACKMT, ROP1L, CMBL, MARH6*
YW	CNV_DUP_3067	DUP	3	85,662,401	86,454,400	1.71E-05	*RL4, NFIA*
	CNV_DEL_4359	DEL	5	20,603,201	20,628,800	1.93E-05	
	CNV_DEL_4552	DEL	5	56,564,001	56,606,400	8.58E-06	*ELOC, SPRY**4**, APOF, IL23A, RDH16, APOF, GP182, CNPY2, RDH16, ATPB, MIP, RDH16, NACAM, RDH16, GLSL, PAN2, STAT**2**, PRI1, H17B6, TIM, BAZ2A, RBMS2*
	CNV_DUP_19581	DUP	21	48,555,201	48,634,400	2.51E-05	*TR10C, TR10D, LIPA1*
	CNV_DUP_20170	DUP	22	38,884,801	39,041,600	3.05E-06	*BHE40, ARL8B, SUMF1, ITPR1*
	CNV_DUP_21558	DUP	23	37,214,401	37,316,800	2.08E-06	

BW, birth weight; WW, weaning weight; ADG, average daily gain; YW, yearling weight; CNV ID, Unique identifier for statistically significant CNVs; Type, the type of significant CNV; Chr, chromosome; Start, the starting position of significant CNV; End, the termination position of significant; *p*-value, the *p*-value of significant CNV; Candidate genes, candidate genes annotated within 300 kb upstream and downstream of significant CNV.

**Table 4:** GO and KEGG functional enrichment of 7 candidate genes for early growth traits of Inner Mongolia cashmere goats.

Trait	Chr	Gene	Significantly enriched pathways
BW	18	*ZN845*	
ADG, WW	13	*SOX15*	cell differentiation、DNA-binding transcription factor activity、RNA polymerase II-specific
	19	*FGF11*	positive regulation of protein phosphorylation、cell differentiation
	13	*GPS2*	regulation of transcription by RNA polymerase II、Human T-cell leukemia virus 1 infection
	19	*DVL2*	regulation of cell population proliferation、Signaling pathways regulating pluripotency of stem cells、positive regulation of protein phosphorylation
YW	5	*SPRY4*	cytosol
	5	*STAT2*	regulation of cell population proliferation、identical protein binding、Osteoclast differentiation

BW, birth weight; WW, weaning weight; ADG, average daily gain; YW, yearling weight; Chr, chromosome; Gene, key candidate gene; Significantly enriched pathways, Functional enrichment of key candidate genes in GO terms and KEGG pathways.

**Figure 4:**
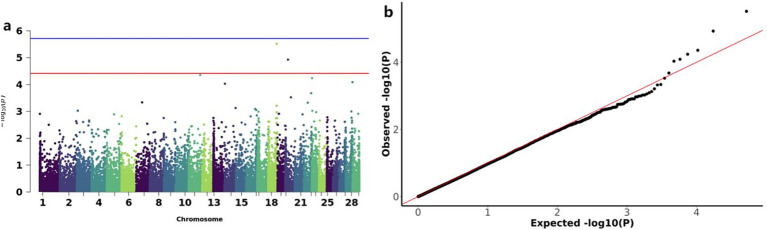
GWAS and significant CNV analysis of BW (birth weight) traits of IMCGs. **(a)** Manhattan diagram of genome-wide association study of birth weight traits; **(b)** Quantile-quantile (Q-Q) plots for genome-wide association studies of birth weight traits; GO, Gene ontology; KEGG, Kyoto Encyclopedia of Genes and Genomes.

The GWAS analysis for BW identified two significant loss CNVs: CNV_DEL_17406 on chromosome 18 and CNV_DEL_18821 on chromosome 20 (Figure 4a). These two CNVs were functionally annotated to four candidate genes, including Zinc finger protein 845 (*ZN845*), Endogenous retrovirus group K member 25 Env polyprotein (*ENK25*), Zinc finger protein 160 (*ZN160*), and Zinc finger protein 208 (*ZN208*) among others (Table 3).

**Figure 5:**
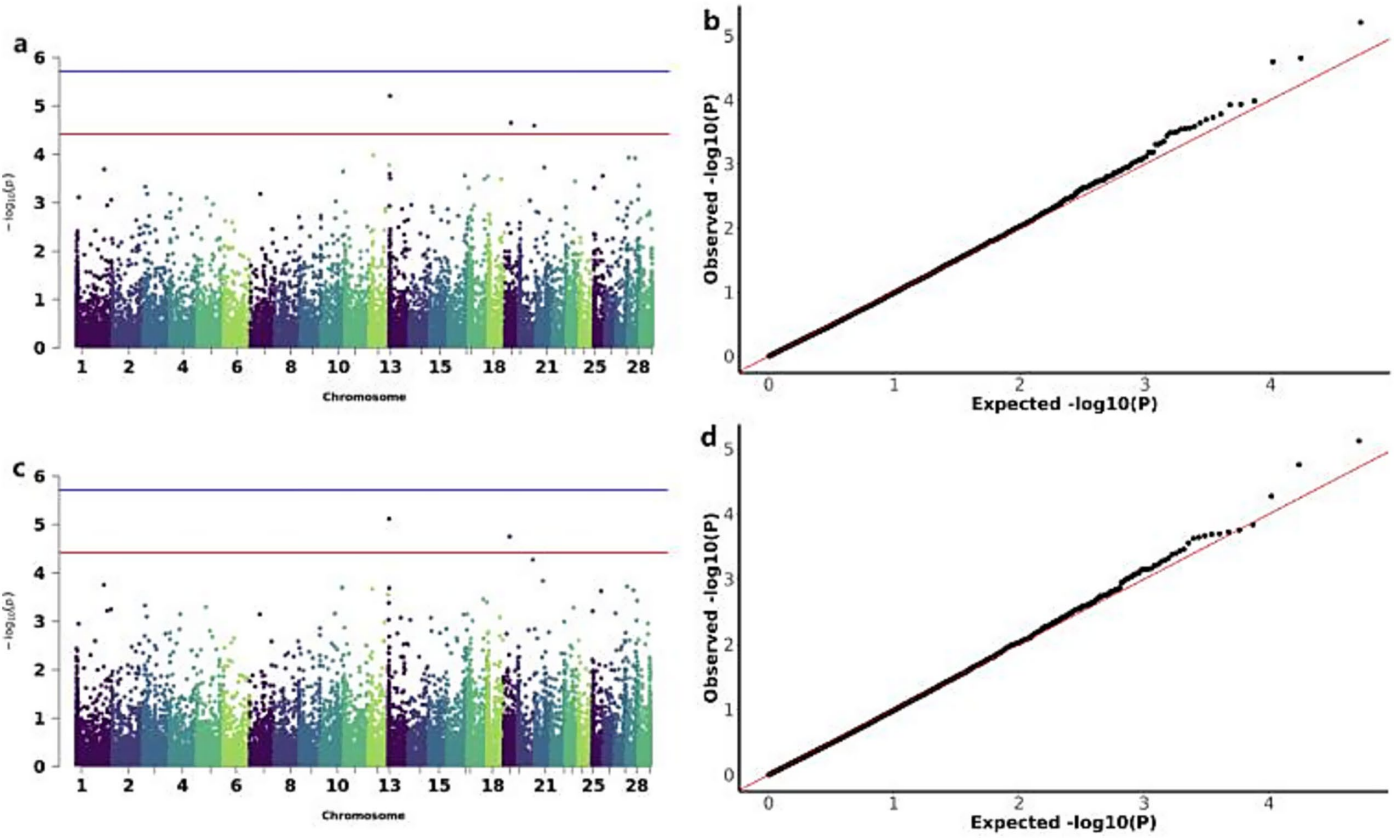
GWAS of ADG (average daily gain) and WW (weaning weight) traits of IMCGs. **(a)** Manhattan diagram of genome-wide association study of average daily gain traits; **(b)** Quantile-quantile (Q-Q) plot of genome-wide association study of average daily gain traits; **(c)** Manhattan diagram of genome-wide association study of weaning weight traits; **(d)** Quantile-quantile (Q-Q) plot of genome-wide association study of weaning weight traits.

The GWAS analysis for ADG and WW identified three significant CNVs: two loss-type CNVs (CNV_DEL_11189 on chromosome 13 and CNV_DEL_17895 on chromosome 19) showing associations with both traits, and one gain-type CNV (CNV_DUP_18956 on chromosome 20) specifically associated with ADG (Figure 5). The overlapping associations of CNV_DEL_11189 and CNV_DEL_17895 with both ADG and WW support the previously reported high genetic correlation between these traits. Functional annotation revealed that these three CNVs encompassed 66 candidate genes, including Transcription factor SOX-15 (*SOX15*) and Thialysine N-epsilon-acetyltransferase (*SAT2*; Table 3).

**Figure 6:**
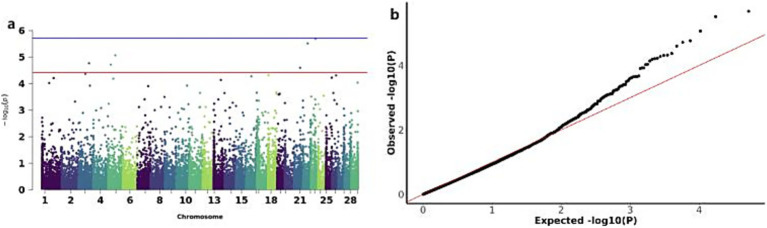
GWAS of YW (yearling weight) traits of IMCGs. **(a)** Manhattan diagram of genome-wide association study of yearling weight traits; **(b)** Quantile-quantile (Q-Q) map of genome-wide association study for yearling weight trait.

The GWAS analysis for YW identified six significant CNVs distributed across chromosomes 3,5,21,22 and 23: CNV_DUP_3067, CNV_DEL_4359, CNV_DEL_4552, CNV_DUP_19581, CNV_DUP_20170, and CNV_DUP_21558 (Figures 6a,b). Functional annotation of these six CNVs revealed their association with 32 candidate genes, including Inositol 1,4,5-trisphosphate-gated calcium channel (*ITPR1*) and ADP-ribosylation factor-like protein 8B (*ARL8B*), among others (Table 3).

### 3.4 Validation of significant CNVs by qPCR

**Figure 7:**
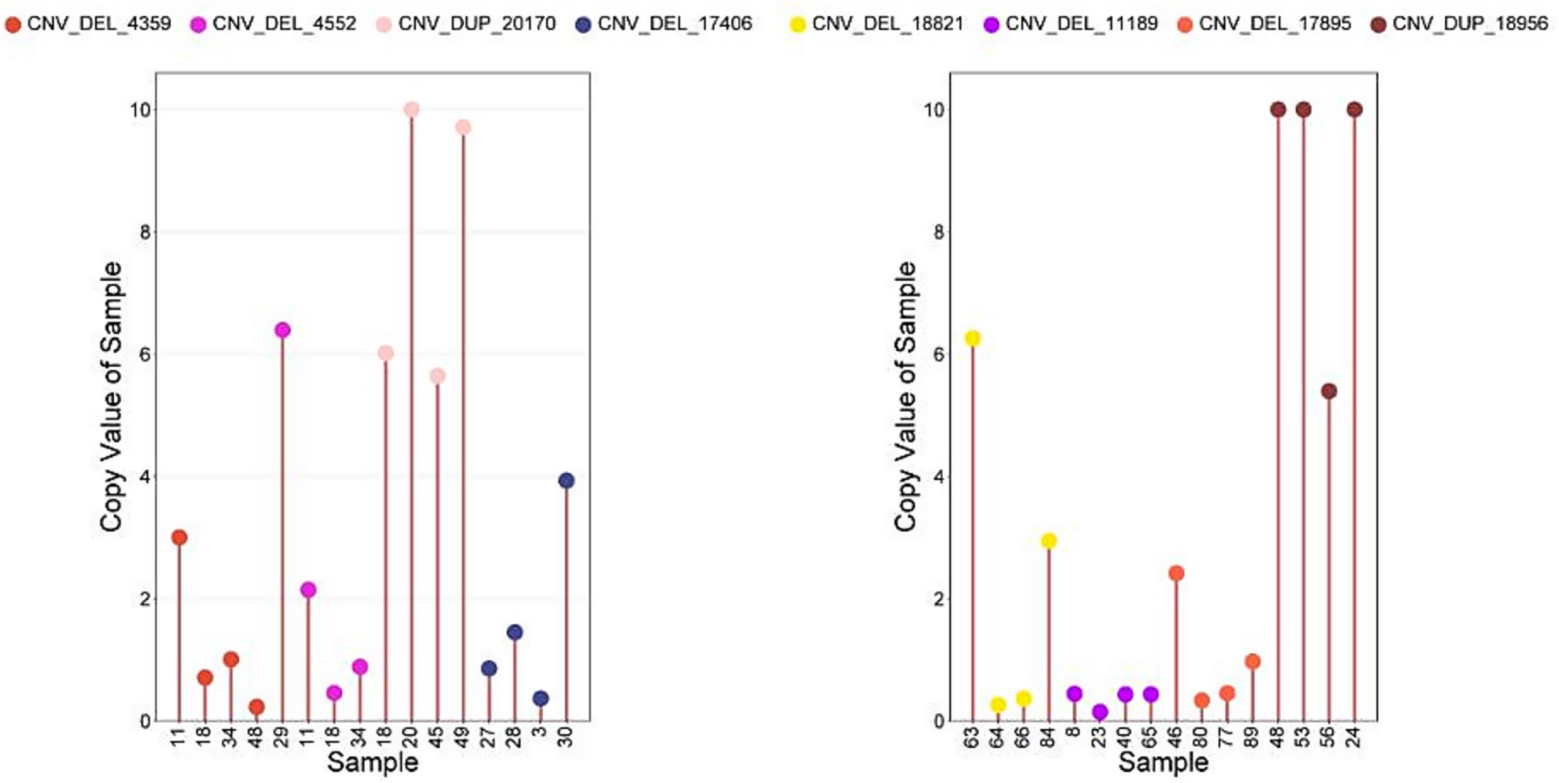
The CNV results were verified by qPCR. Sample, Individuals performing copy number detection; Copy Value of Sample, the copy number of the experimental individual; Copy Value: Experimental samples (values >10 truncated at 10); Control samples (default copy number = 2).

The qPCRs were performed to verify 8 CNVs in 32 samples. As shown in Figure 4, more than 78.0% of the results are consistent with the type of CNV predicted using CNVnator (Figure 7).

**Figure 8:**
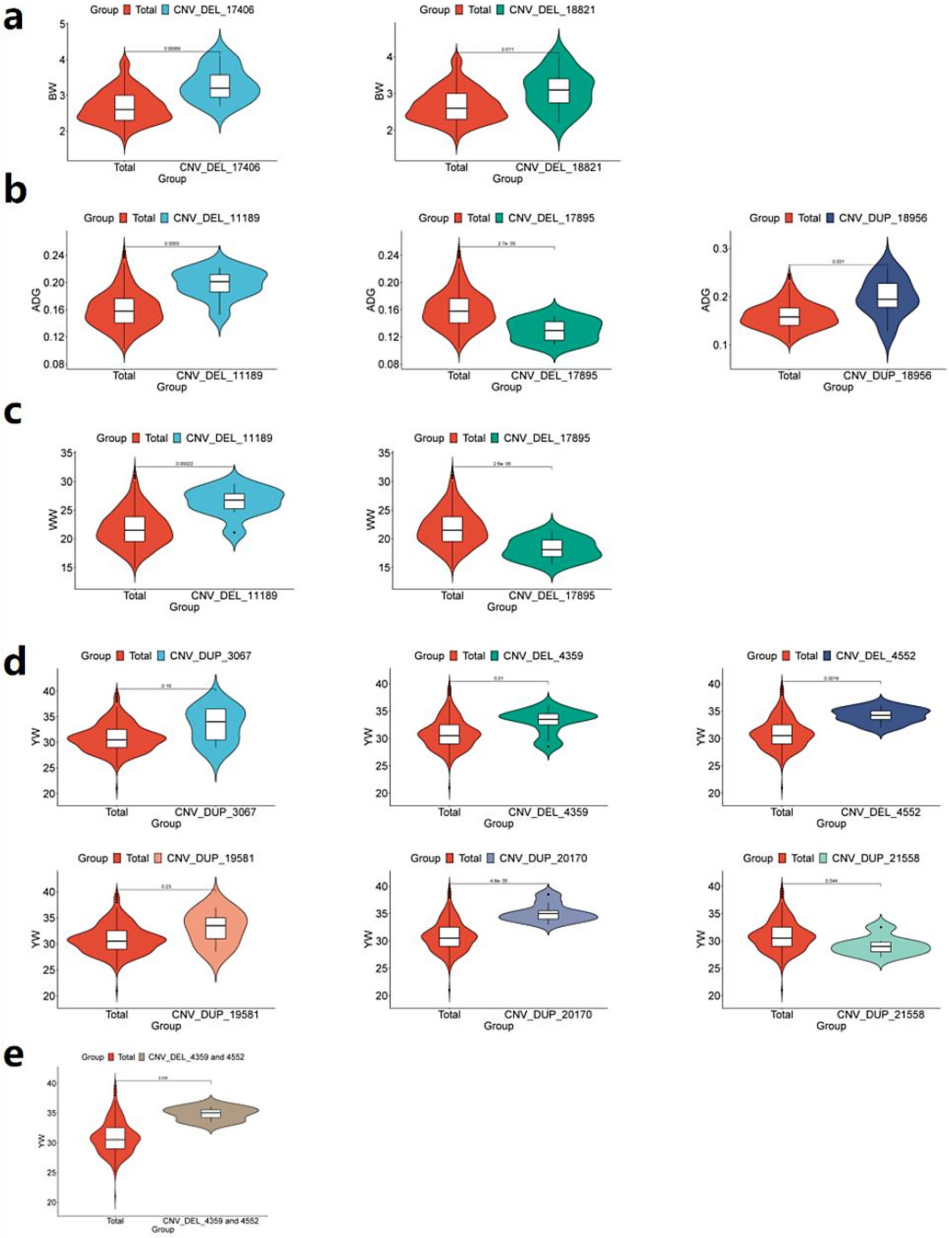
Phenotypic comparison between individuals with significant CNVs and IMCGs populations. **(a)** Phenotypic analysis of BW (birth weight) traits of individuals containing CNV_DEL_17406 or CNV_DEL_18821; **(b)** Phenotypic analysis of ADG (average daily gain) traits in individuals containing CNV_DEL_11189 or CNV_DEL_17895 or CNV_DUP_18956; **(c)** Phenotypic analysis of WW (weaning weight) trait in individuals with CNV_DEL_11189 or CNV_DEL_17895; **(d)** Phenotypic analysis of YW (yearling weight) traits of individuals containing CNV _DUP_3067 or CNV_DEL_4359 or CNV_DEL_4552 or CNV_DUP_19581 or CNV_DUP_20170 or CNV_DUP_21558; **(e)** Phenotypic analysis of YW (yearling weight) traits containing CNV_DEL_4359 and CNV_DEL_4552 individuals; Total, phenotypic value of Inner Mongolia cashmere goat population; unit: kg.

Comparative analysis revealed that individuals carrying either CNV_DEL_17406 or CNV_DEL_18821 exhibited significantly higher average BW (*p < 0.05*) compared to the overall IMCG population mean of 2.69 kg (Figure 8a). This finding suggests a potential positive association between these CNVs and increased birth weight in the IMCG population.

Comparative analysis demonstrated significant phenotypic effects: individuals carrying CNV_DEL_11189 or CNV_DUP_18956 exhibited higher average ADG (*p < 0.05*) than the population mean (0.16 kg), while those with CNV_DEL_17895 showed lower ADG (*p <* 0.05; Figure 8b). Similarly, for WW traits, CNV_DEL_11189 carriers displayed significantly higher WW (*p <* 0.05) than the population mean (21.90 kg), whereas CNV_DEL_17895 carriers showed reduced WW (*p <* 0.05; Figure 8c).

Comparative phenotypic analysis demonstrated distinct effects of these CNVs on YW: individuals carrying CNV_DEL_4359, CNV_DEL_4552, or CNV_DUP_20170 exhibited significantly higher YW (*p <* 0.05) than the population mean (31.21 kg). While CNV_DUP_3067 and CNV_DUP_19581 carriers showed elevated YW compared to the population mean, these differences did not reach statistical significance. In contrast, individuals with CNV_DUP_21558 displayed significantly lower YW (*p <* 0.05) than the population average (Figure 8d). Notably, CNV_DEL_4359 and CNV_DEL_4552 showed particularly strong positive associations with YW, with carriers exhibiting significantly higher weights than the population mean (Figure 8e).

Comparative analysis using independent samples revealed significant phenotypic divergence (*p <* 0.05) between CNV carriers and the Inner Mongolia Cashmere Goats (IMCGs) population baseline in 9 out of 11 candidate copy number variations (CNVs) associated with early growth traits. While two CNV loci (CNV_DEL_17406 and CNV_DUP_20170) showed non-significant *p*-values (0.19 and 0.23, respectively), their effect sizes (Cohen’s d > 0.4) demonstrated meaningful biological divergence from population means. This validation framework achieved 81.8% concordance between GWAS-identified loci and measurable phenotypic effects, with the remaining variants showing directional consistency in trait modulation, thereby providing orthogonal validation for the GWAS findings.

### 3.5 Functional analysis of genes associated with trait-related CNVs

**Figure 9:**
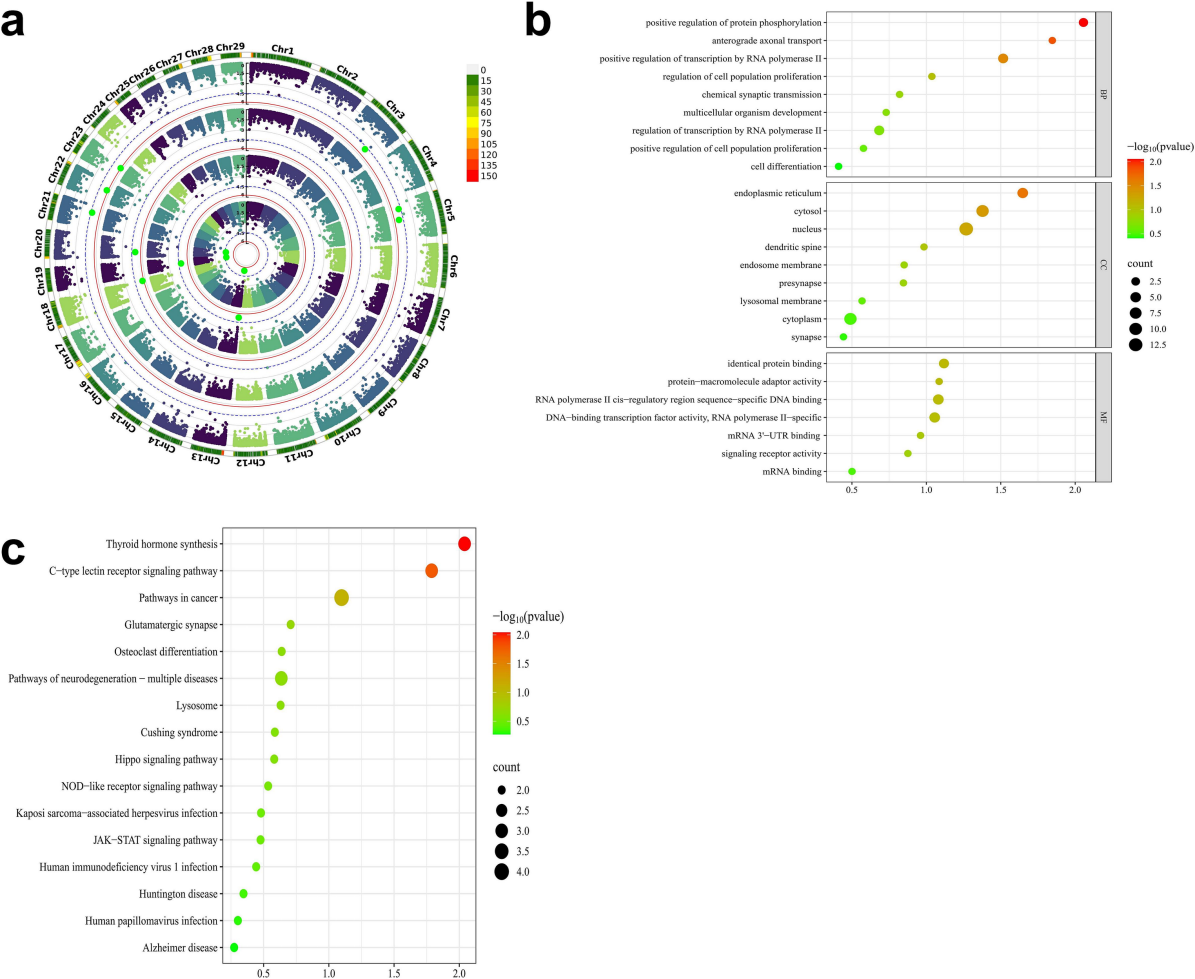
GO and KEGG functional enrichment maps of key candidate genes for early growth traits of Inner Mongolia cashmere goats. **(a)** The Manhattan diagram of the GWAS results of the early growth traits of Inner Mongolia cashmere goats; **(b)** The GO functional enrichment map of the key candidate genes; **(c)** The KEGG pathway enrichment map of the key candidate genes; GO, Gene ontology; KEGG, Kyoto Encyclopedia of Genes and Genomes.

Genomic annotation was performed on genes overlapping with the 11 significant CNVs, including their upstream and downstream 300 kb flanking regions. Based on the Ensembl annotation of the *Capra hircus* reference genome (Genome assembly ASM4082201v1), a total of 91 genes were identified. To elucidate the functional relevance of these genes to early growth traits in IMCGs, comprehensive enrichment analyses were conducted using Kyoto Encyclopedia of Genes and Genomes (KEGG) pathways and Gene Ontology (GO) terms. The gene set enrichment analysis revealed several functionally relevant terms associated with early growth traits in IMCGs. Specifically, the analysis identified 20 significant GO terms, comprising 6 cellular component terms, 7 biological process terms, and 7 molecular function terms. KEGG demonstrated that these genes are predominantly involved in crucial biological processes, including cellular development, hormonal biosynthesis, protein phosphorylation, and related metabolic pathways (Figure 9).

Through integrative analysis combining data from the GeneCards database and published literature, we identified several genes implicated in key biological pathways and processes. Seven candidate genes were selected from this analysis based on their overlap with significant CNVs and enrichment in gene set analysis (Table 4). The candidate gene analysis revealed *ZN845* was as the primary candidate for BW, while *SOX15, FGF11, GPS2*, and *DVL2* emerged as candidate genes for WW and ADG. For YW, *SPRY4* and *STAT2* were identified as potential candidate genes. These candidate genes associated traits are comprehensively summarized in Table 4.

Functional enrichment analysis revealed distinct biological roles for the identified candidate genes. For BW traits, *ZN845* did not exhibit significant enrichment in any specific pathways within our analysis framework. For ADG and WW traits, the *SOX15* demonstrated significant associations with critical cellular processes, including cell differentiation, DNA-binding transcription factor activity, and RNA polymerase II-specific regulation. *FGF11* was prominently enriched in pathways governing protein phosphorylation modulation and cellular differentiation processes. *GPS2* showed specific involvement in transcriptional regulation mediated by RNA polymerase II, particularly in the context of human T-cell leukemia virus 1 infection. *DVL2* was significantly enriched in multiple pathways, including the regulation of cell population proliferation, signaling pathways controlling stem cell pluripotency, and positive regulation of protein phosphorylation. For YW traits, *STAT2* was associated with fundamental biological processes such as cell proliferation, same-protein binding interactions, and the regulation of osteoclast differentiation. *SPRY4* is significantly enriched in cell solutes (Table 4).

## 4 Discussion

With the rapid development of bioinformatics, the number of SNPs that significantly affect complex traits through GWAS analysis is increasing. However, many SNPs can only explain the heritability of some complex traits, which is called ‘missing heritability (30, 31). CNV is a widespread variation phenomenon in genome genetic variation and an important part of human, animal and plant genomes. GWAS analysis using CNV may explain the genetic variation of complex traits that cannot be explained by some SNPs (32). Early growth traits (BW, ADG, WW, YW) are important traits of Inner Mongolia Cashmere Goats throughout the feeding cycle (2). Therefore, it is of great significance for the breeding and feeding of cashmere goats to explore the molecular regulation mechanism of early growth traits of Inner Mongolia cashmere goats and the key candidate genes affecting early growth traits of cashmere goats. Although there have been many SNP-based GWAS studies to identify the key genes of weight traits in goats (8, 33). However, there are relatively few studies on GWAS analysis of body weight traits of cashmere goats based on CNV to determine the key genes affecting body weight traits of cashmere goats. Therefore, this study is the first to use the whole genome resequencing of Inner Mongolia Cashmere Goats to seen the CNV of Inner Mongolia Cashmere Goats population, and perform GWAS analysis of BW, ADG, WW, and YW to mine key candidate genes. In this study, CNVnator was used to detect CNV in high-depth whole-genome resequencing data (13.41 Tb) of 461 Inner Mongolia cashmere goats based on Read Depth method and 800 bp sliding window. The detection of CNV using resequencing data usually requires the sequencing depth of sequencing data to be at least 5 × and above (34). The average sequencing depth of resequencing data used in this study was 9.46×, which met the requirements of resequencing data to detect CNV. A total of 315,891 CNVs were detected in this study. After deleting CNVs with abnormal length and low frequency (1%), 26,003 CNVs were finally retained for CNVR merging and GWAS analysis. Among them, 10,216 (39.3%) were gain-type CNVs and 15,787 (60.7%) were loss-type CNVs. The number of loss-type CNVs was greater than that of gain CNVs, which was consistent with the results of other studies on CNV detection in cattle, sheep and pigs (12, 35, 36). Among the 32 experimental samples subjected to qPCR analysis, target CNVs were successfully detected in 25 samples, demonstrating a detection frequency of 78.0% (25/32). Notably, each target CNV was detected in at least two experimental individuals, ensuring the reliability of our findings. Furthermore, analysis of the seven experimental individuals that did not show target CNV detection revealed that five of these individuals exhibited copy number variations (CNVs) different from the normal diploid state (copy number ≠ 2). This observation suggests the presence of genomic structural variations in the target CNV region, thereby providing additional evidence supporting the authenticity and reliability of the CNVs detected in this study.

Based on the GWAS results, we identified 11 CNVs significantly associated with BW, ADG, WW and YW. Comparative analysis between the phenotypes of individuals carrying significant CNVs and the overall population revealed distinct patterns of CNV effects. For the BW trait, two significant CNVs (CNV_DEL_17406 and CNV_DEL_18821) exhibited a gain-of-function effect in IMCG.

Furthermore, we identified two CNVs (CNV_DEL_11189 and CNV_DEL_17895) that were shared between ADG and WW traits, along with one ADG-specific CNV (CNV_DUP_18956). Notably, while CNV_DUP_18956 did not reach genome-wide significance for WW (*p =* 5.34 × 10^−5^), its *p*-value was remarkably close to the significance threshold (*p <* 3.85 × 10^−5^). Functional analysis showed that CNV_DEL_11189 exerted a gain effect on ADG and WW in IMCG, whereas CNV_DEL_17895 displayed an inhibitory effect on these traits. The ADG-specific CNV_DUP_18956 demonstrated a gain effect on ADG in IMCG. Regarding YW, we detected six significant CNVs (CNV_DUP_3067, CNV_DEL_4359, CNV_DEL_4552, CNV_DUP_19581, CNV_DUP_20170, and CNV_DUP_21558). Among these, CNV_DUP_21558 showed an inhibitory effect on YW traits in IMCG, while the remaining five CNVs exhibited gain effects on YW.

Following the annotation methodology described by Xin et al. (37), we performed comprehensive gene annotation within 300 kb upstream and downstream regions of the 11 identified CNVs, identifying a total of 91 genes. Subsequent GO and KEGG pathway enrichment analyses revealed that 37 of these genes were significantly enriched in various biological functions and pathways. Through integration of previous research findings with GO and KEGG enrichment results, we identified seven key candidate genes potentially influencing early growth traits in IMCGs: *ZN845*, *SOX15*, *FGF11*, *GPS2*, *DVL2*, *SPRY4*, and *STAT2*. Considering that weight gain in animals is closely associated with muscle development, fat deposition, and obesity (3), Functional enrichment analysis was conducted for these seven candidate genes. The results demonstrated their predominant involvement in crucial biological pathways, particularly cell proliferation and energy metabolism.

Among these genes, zinc finger protein 845 (*ZN845*), located within the CNV_DEL_17406 region on chromosome 18, emerged as particularly relevant to body weight. As a member of the zinc finger protein family, *ZN845* represents a crucial class of transcription factors that play pivotal roles in various biological processes, including DNA recognition, RNA packaging, transcriptional activation, apoptosis regulation, protein folding and assembly, and lipid binding. Previous studies have established its critical functions in plant stress resistance and abiotic stress responses (29, 38), as well as its regulatory role in animal muscle development. Our findings strongly suggest that *ZN845* serves as a key candidate gene influencing early growth traits in cashmere goats.

The transcription factor SOX-15 (*SOX15*), located within the CNV_DEL_17895 region on chromosome 19, represents the sole member of group G in the Sry-related high-mobility-group (*HMG*) box (*SOX*) gene family. This gene demonstrates significant associations with ADG and WW, playing crucial roles in multiple biological processes, including male gonad development, striated muscle tissue formation, myoblast proliferation, and skeletal muscle regeneration. Experimental evidence from Ito et al. (39) demonstrated that mice with defective *SOX15* genes exhibited significantly delayed skeletal muscle regeneration, highlighting the gene’s critical role in murine muscle development. Furthermore, Kayo Yamada et al. (40) identified substantial *SOX15* expression in murine placental tissue, suggesting its significant impact on placental development in mammals. These collective findings from both muscle and placental development studies strongly indicate that the *SOX15* gene serves as a key candidate gene influencing early growth traits in Inner Mongolia cashmere goats.

Fibroblast growth factor 11 (*FGF11*), co-localized with *SOX15* in the CNV_DEL_17895 region of chromosome 19, represents a crucial member of the intracellular fibroblast growth factor family, primarily involved in nervous system development and function in animals (41). Experimental evidence from Zhao et al. (42) demonstrated that hypothalamic injection of RNA virus to inactivate *FGF11* in mice fed a high-fat diet resulted in significant body weight reduction, decreased fat synthesis rates, and increased brown fat thermogenesis, highlighting the gene’s substantial impact on adipocyte synthesis. Further supporting this, Li et al. (43) observed that *FGF11* knockout mice exhibited reduced expression of peroxisome proliferator-activated receptor gamma (*PPARcγ*) gene, accompanied by decreased rates of adipocyte proliferation and differentiation. Notably, restoration of *FGF11* expression normalized *PPARcγ* levels and accelerated adipocyte proliferation and differentiation. Recent findings by Jiang et al. (44) in goat revealed that *FGF11* specifically regulates brown adipocyte differentiation and thermogenesis, while showing no significant effect on white adipocyte proliferation and differentiation. These collective findings underscore the dual role of *FGF11* in both adipocyte proliferation/differentiation and thermoregulation in goats. *FGF11* was identified as a key candidate gene for early growth traits in Inner Mongolia cashmere goats, based on its established roles in adipocyte regulation and cold resistance.

The G protein pathway suppressor 2 (*GPS2*) gene, co-localized within the CNV_DEL_17895 region on chromosome 19, plays a crucial role in multiple biological processes including inflammation regulation, adipocyte differentiation, and lipid metabolism. Experimental studies by Justin et al. (45) demonstrated that *GPS2* knockout (*GPS2* AKO) mice fed a high-fat diet exhibited significantly increased susceptibility to inflammation and disrupted fat synthesis processes, accompanied by marked adipocyte proliferation. Further supporting these findings, Drareni et al. (46) established a connection between *GPS2* and the expansion of hypertrophic white adipose tissue in humans. Their observations of *GPS2* AKO mice revealed adipocyte hypertrophy, inflammation, and mitochondrial dysfunction under conditions of energy excess, providing compelling evidence for *GPS2’s* significant role in adipocyte differentiation and inflammatory responses. These collective findings from both murine and human studies strongly suggest that the *GPS2* gene serves as a key candidate gene influencing early growth traits in Inner Mongolia cashmere goats, particularly through its regulatory effects on adipocyte differentiation and metabolic processes.

Segment polarity protein disheveled homolog *DVL-2* is also located in the CNV_DEL_17895 region of Chr19, which is involved in cell proliferation, protein phosphorylation and osteoblast differentiation (47). Yamaguchi et al. found that the body ‘s Stau1 protein negatively regulates the myogenesis of *C2C12* myoblasts by binding to the mRNA 3’untranslated region (UTR) of the *DVL2* gene. It shows that *DVL2* has an inhibitory effect on myogenesis (48). The results from this study indicated that *DVL2* is a key candidate gene for early growth traits of Inner Mongolia cashmere goats.

SPRY domain-containing protein 4 (*SPRY4*) located in the CNV_DEL_4552 region of Chr5 is a protein-coding gene of the Spry family, which is related to YW and participates in biological processes such as cell proliferation, migration, inflammation, oxidative stress, apoptosis and organ development (49). Li et al. (50) found that *SPRY4* was positively correlated with adipogenic differentiation of human mesenchymal stem cells (MSC). *In vivo* and *in vitro* experiments confirmed that *SPRY4* promoted hAMSC adipogenesis through MEK-ERK1/2 pathway, indicating that *SPRY4* was related to adipogenesis. It indicated that *SPRY4* gene was a key candidate gene for early growth traits of Inner Mongolia cashmere goats.

Signal transducer and activator of transcription 2 (*STAT2*), also located in CNV_DEL_4552 region of Chr5, is involved in cell proliferation, protein phosphorylation and type I interferon mediated signaling pathway (51). Yang et al. found that circCAPRIN1 promotes lipid synthesis by enhancing Acetyl-CoA carboxylase 1 (*ACC1*), and further analysis found that circCAPRIN1 directly binds to signal transduction and *STAT2* gene to activate ACC1 transcription, thereby regulating lipid metabolism, indicating that *STAT2* gene is related to lipid synthesis in organisms (52). The results from this study indicated that *STAT2* is a key candidate gene for early growth traits of Inner Mongolia cashmere goats.

The body weight is related to animal muscle development, fat deposition and obesity. The key candidate genes of early growth traits of Inner Mongolia cashmere goats identified in this study are related to animal fat differentiation and muscle development.

## 5 Conclusion

The first CNV landscape were constructed in Inner Mongolia Cashmere Goats in this study. The 11 significant CNVs and 7 key candidate genes (*ZN845, SOX15, FGF11, GPS2, DVL2, SPRY4, STAT2*) related to early growth traits were identified. Among them, *FGF11* regulates adipogenesis, while the other genes are novel regulatory factors for muscle or fat development in this breed. It is inferred that these genes may be functional targets influencing the early growth and development of Inner Mongolia cashmere goats, providing a reference for subsequent molecular breeding efforts.

## Data Availability

The original contributions presented in the study are publicly available. This data can be found here: NCBI SRA repository, accession number PRJNA1332427, https://www.ncbi.nlm.nih.gov/sra/PRJNA1332427. The phenotypic datasets presented in this article are not readily available due to confidentiality purposes.

## Footnotes

^1^ https://www.ncbi.nlm.nih.gov/datasets/genome/GCA_040822015.1/

^2^ www.ensembl.org/biomart/martview
