# Supplementary material for: Genome-wide association study of copy number variation and early growth traits in inner Mongolian cashmere goats
Source: Front Vet Sci. 2025 Oct 17;12:1651622. doi: 10.3389/fvets.2025.1651622 (PMC12576916; doi:10.3389/fvets.2025.1651622)
Supplement: Supplementary file 1 [file Table_1.docx]

Genome-Wide Association Study of Copy Number Variation and Early Growth Traits in Inner Mongolian Cashmere Goats

**Yifan Liu^1,2,3^, Haijiao Xi^1,2,3^, Qi Xu^1,2,3^, Bohan Zhou^1,2,3^, Jinquan Li^1,2^, Rui Su^1,2,3^, Qi Lv^1,2,3^, Yanjun Zhang^1,2,3^, Ruijun Wang^1,2,3^, Zhiying Wang^1,2,3^,***

Table S1. qPCR primer and probe sequence information

| CNVs | Chr | Forward（5'-3'） | Reverse（5'-3'） |
| --- | --- | --- | --- |
| CNV_DEL_4359 | 5 | ACTGCCTGATACTGAGTTTCCA | CACAAATGTTTACAGCAGCGT |
| CNV_DEL_4552 | 5 | TGCAAGGAGGAAGCTAGACC | TGAGCAGAGTTCCATGTCCT |
| CNV_DUP_20170 | 22 | ATCTGGTGGGAGAGTTTGCA | GAGGGGAGGGGACAGTTATG |
| CNV_DEL_17406 | 18 | TCCTCCGAGTGTTCAAACCA | TCCCTATGTGTTTACGGTCT |
| CNV_DEL_18821 | 20 | AAGGAGACAAAGGAGGTGGG | TCAGCAGGGAGAGTGTTTCC |
| CNV_DEL_11189 | 13 | GACAGTGCTGCTACAACTCG | TCCTCTCCAGTGCTGACATG |
| CNV_DEL_17895 | 19 | GCCAGGCTGTGTAAGAAGTG | GGTGTCTTGCGTTGCTTAGG |
| CNV_DUP_18956 | 20 | TGCATGTCTGTGTGTGTGAC | TCCAACCCAGGGATTGAACC |
| ACTB | 1 | CCCTGGAGAAGAGCTACGAG | TAGTTTCGTGAATGCCGCAG |

Abbreviations: CNVs= verified CNV；Chr= chromosome; Forward（5'-3'）= forward primer sequence Reverse（5'-3'）= reverse primer sequence.

Table S2. CNVR descriptive statistics

| Chr | length (kb) | CNVR counts | Length of CNVR (kb) | Coverage (%) | Max size (kb) | Average size (kb) | Min size (kb) |
| --- | --- | --- | --- | --- | --- | --- | --- |
| 1 | 163599655 | 344 | 66064800 | 40.38 | 5278400 | 192048.84 | 1600 |
| 2 | 138024171 | 267 | 50842400 | 36.84 | 4587200 | 190420.97 | 1600 |
| 3 | 122269964 | 276 | 44209600 | 36.16 | 4652000 | 160179.71 | 1600 |
| 4 | 123500297 | 247 | 39647200 | 32.10 | 2583200 | 160514.98 | 1600 |
| 5 | 120353402 | 254 | 36261600 | 30.13 | 4760800 | 142762.20 | 1600 |
| 6 | 118907578 | 217 | 35036800 | 29.47 | 5974400 | 161459.91 | 1600 |
| 7 | 108857206 | 245 | 36660800 | 33.68 | 3284000 | 149635.92 | 1600 |
| 8 | 117324300 | 216 | 37060000 | 31.59 | 4484000 | 171574.07 | 1600 |
| 9 | 92782956 | 213 | 25237600 | 27.20 | 1817600 | 118486.38 | 1600 |
| 10 | 109890027 | 266 | 41044000 | 37.35 | 1899200 | 154300.75 | 1600 |
| 11 | 108503269 | 225 | 43721600 | 40.30 | 5149600 | 194318.22 | 1600 |
| 12 | 91085198 | 123 | 42136000 | 46.26 | 4960000 | 342569.11 | 1600 |
| 13 | 89647956 | 139 | 40302400 | 44.96 | 5753600 | 289945.32 | 1600 |
| 14 | 95224248 | 181 | 27188000 | 28.55 | 3641600 | 150209.94 | 1600 |
| 15 | 82415014 | 193 | 29837600 | 36.20 | 2164000 | 154598.96 | 1600 |
| 16 | 91491535 | 147 | 38597600 | 42.19 | 6874400 | 262568.71 | 1600 |
| 17 | 90066741 | 158 | 44683200 | 49.61 | 6545600 | 282805.06 | 1600 |
| 18 | 74692390 | 139 | 45996000 | 61.58 | 4545600 | 330906.47 | 1600 |
| 19 | 63059135 | 111 | 41355200 | 65.58 | 9339200 | 372569.37 | 1600 |
| 20 | 78028267 | 118 | 25727200 | 32.97 | 5857600 | 218027.12 | 1600 |
| 21 | 72404257 | 144 | 27694400 | 38.25 | 2244000 | 192322.22 | 1600 |
| 22 | 67135385 | 109 | 29719200 | 44.27 | 6012800 | 272653.21 | 1600 |
| 23 | 59408587 | 102 | 30975200 | 52.14 | 5171200 | 303678.43 | 1600 |
| 24 | 63378952 | 125 | 20003200 | 31.56 | 2479200 | 160025.60 | 1600 |
| 25 | 48079060 | 75 | 26138400 | 54.37 | 6067200 | 348512.00 | 1600 |
| 26 | 51813540 | 104 | 17853600 | 34.46 | 6302400 | 171669.23 | 1600 |
| 27 | 53382197 | 88 | 18271200 | 34.23 | 3536000 | 207627.27 | 1600 |
| 28 | 54948720 | 91 | 26006400 | 47.33 | 4981600 | 285784.62 | 1600 |
| 29 | 55425393 | 97 | 27160800 | 49.00 | 5890400 | 280008.25 | 1600 |

Abbreviations: Chr = chromosome；Length: the length of the chromosome; CNVR counts= the number of CNVR; Length of CNVR= the length of CNVR; Coverage= CNVR length / chromosome length*100%; Max size = the maximum value of CNVR；Average size= the average value of CNVR；Min size= the minimum value of CNVR.

**S3. Custom analysis scripts**

**1. Genotyping Workflow**

**# 1.1 Raw data QC**

$ fastqc -o /output_dir/ /input_dir/sample.fq.gz

**# 1.2 Adapter trimming & quality filtering**

$ trimmomatic PE -phred33 \

-trimlog sample.log \

sample_R1.fq.gz sample_R2.fq.gz \

sample_clean_R1.fq.gz sample_unpaired_R1.fq.gz \

sample_clean_R2.fq.gz sample_unpaired_R2.fq.gz \

ILLUMINACLIP:TruSeq3-PE.fa:2:30:10 \

SLIDINGWINDOW:5:20 LEADING:5 TRAILING:5 MINLEN:50

**# 1.3 Alignment to reference genome**

$ bwa mem -t 8 -R '@RG\tID:sample\tPL:ILLUMINA\tLB:library\tSM:sample' \

/ref_genome/goat.fasta \

sample_clean_R1.fq.gz sample_clean_R2.fq.gz \

| samtools view -@ 4 -Sb - > sample.bam

**# 1.4 Sorting & deduplication**

$ samtools sort -@ 8 -m 4G -O BAM -o sample.sorted.bam sample.bam

$ samtools markdup -@ 8 -r sample.sorted.bam sample.final.bam

**2. CNV Detection (CNVnator)**

**# 2.1 Read extraction from BAM**

$ cnvnator -root 1.root -chrom Chr1 ... Chr29 -tree 1..bam -genome genome.fa

**# 2.2 Histogram generation**

$ cnvnator -root 1.root -chrom Chr1 ... Chr29 -bin_size [-d dir]

**# 2.3 Statistical calculation**

$ cnvnator -root 1.root -chrom Chr1 ... Chr29 -stat bin_size

**# 2.4 Signal partitioning**

$ cnvnator -root 1.root -chrom Chr1 ... Chr29 -partition bin_size [-ngc]

**# 2.5 CNV calling**

$ cnvnator -root 1.root -chrom Chr1 ... Chr29 -call bin_size [-ngc]

**3. GWAS Analysis (PLINK)**

$ plink --bfile imcg_genotypes \

--pheno growth_traits.txt \

--pheno-name ADG \

--linear hide-covar \

--allow-extra-chr \

--chr-set 29 \

--covar covariates.txt \

--covar-name sex,age \

--out gwas_adg_results
